# Supplementary material for: Kinesthetic motor-imagery training improves performance on lexical-semantic access
Source: PLoS One. 2022 Jun 24;17(6):e0270352. doi: 10.1371/journal.pone.0270352 (PMC9232155; doi:10.1371/journal.pone.0270352)
Supplement: Attachment — Submitted filename: Review_Plos_One.docx [file pone.0270352.s001.docx]

I find the paper very interesting and well written. In two different experiments, it has been shown the impact of motor imagery on lexical-semantic access, specifically it has been demonstrated that a kinesthetic motor imagery training improves language comprehension.

Please find below my comments:

Introduction

The introduction lack of some references about behavioural and neurophysiological studies on the cross-talk between action and language, which sometimes are in contrast. Indeed some studies have highlighted a facilitation, some others an interference effect in case of congruency between the meaning of the sentence and the performed action.

I suggest to look at:

Scorolli, C., & Borghi, A. M. (2007). Sentence comprehension and action: Effector specific modulation of the motor system. *Brain research*, *1130*, 119-124.

Gianelli, C., & Dalla Volta, R. (2015). Does listening to action-related sentences modulate the activity of the motor system? Replication of a combined TMS and behavioral study. *Frontiers in psychology*, *5*, 1511.

Sato, M., Mengarelli, M., Riggio, L., Gallese, V., & Buccino, G. (2008). Task related modulation of the motor system during language processing. Brain and Language, 105(2), 83–90.

Miller, J., Brookie, K., Wales, S., Wallace, S., & Kaup, B. (2018). Embodied cognition: Is activation of the motor cortex essential for understanding action verbs?. *Journal of Experimental Psychology: Learning, Memory, and Cognition*, *44*(3), 335.

Chersi, F., Thill, S., Ziemke, T., & Borghi, A. M. (2010). Sentence processing: linking language to motor chains. *Frontiers in neurorobotics*, *4*, 4.

The authors does not mention any reference concerning the relation between action and abstract/concrete language, although such dichotomy is matter of investigation of the studies and formulate some predictions.

Please look at:

Scorolli, C., Binkofski, F., Buccino, G., Nicoletti, R., Riggio, L., & Borghi, A. M. (2011). Abstract and concrete sentences, embodiment, and languages. *Frontiers in Psychology*, *2*, 227.

Ghandhari, M., Fini, C., Da Rold, F., & Borghi, A. M. (2020). Different kinds of embodied language: A comparison between Italian and Persian languages. *Brain and Cognition*, *142*, 105581.

Desai, R. H., Conant, L. L., Binder, J. R., Park, H., & Seidenberg, M. S. (2013). A piece of the action: modulation of sensory-motor regions by action idioms and metaphors. *NeuroImage*, *83*, 862-869.

Sakreida, K., Scorolli, C., Menz, M. M., Heim, S., Borghi, A. M., & Binkofski, F. (2013). Are abstract action words embodied? An fMRI investigation at the interface between language and motor cognition. *Frontiers in human neuroscience*, 125.

Scorolli, C., Binkofski, F., Buccino, G., Nicoletti, R., Riggio, L., & Borghi, A. M. (2011). Abstract and concrete sentences, embodiment, and languages. *Frontiers in Psychology*, *2*, 227.

Sample size:

The authors does not justify with any reference to the literature or power analysis the sample size of the both the experiments. Please provide a justification.

Could you please report and translate the items in English of KIVQ-10?

Why did you opt for a between-subjects design in the first experiment, instead of a within-subject design in which participant would have performed in different days?

Lines 214: “presented” repeated twice

Data Analysis

Could you please report the number of RTs discarded in both the experiments?

Could you explain the Cook D criteria?

Did you adopt as criteria the stepwise procedure in your models selection? Starting with the model with the more complex random structure? See the following reference:

Barr, D. J., Levy, R., Scheepers, C., & Tily, H. J. (2013). Random effects structure for confirmatory hypothesis testing: Keep it maximal. *Journal of memory and language*, *68*(3), 255-278.

Results & Discussion

I see that in the first experiment you got that participants were faster with abstract as compared with concrete verbs. Why in your opinion this is the case? Could you please argument more about that in the discussion?

Did you consider the influence of the culture in explaining your results? Were all your participants native French? Was someone bilingual?

I recommend to consider these factors as it is suggested in Ghandhari, M., Fini, C., Da Rold, F., & Borghi, A. M. (2020). Different kinds of embodied language: A comparison between Italian and Persian languages. *Brain and Cognition*, *142*, 105581.

It might depend on the level of integration between language and action, it might depend on the perceptual strength associated with the verbs.

One possibility is that abstract verbs “might occupy less the sensorimotor system “ and their categorization might be favoured, to better clarify , I mean that it might be possible that a congruency between action and concrete verbs is expressed by a interference effect instead of facilitation.

Finally, Here in you can find a broad perspective on the relation between the motor system and the language, especially the abstract one. Mazzuca, C., Fini, C., Michalland, A. H., Falcinelli, I., Da Rold, F., Tummolini, L., & Borghi, A. M. (2021). From affordances to abstract words: The flexibility of sensorimotor grounding. *Brain Sciences*, *11*(10), 1304.
