# Supplementary material for: Kinesthetic motor-imagery training improves performance on lexical-semantic access
Source: PLoS One. 2022 Jun 24;17(6):e0270352. doi: 10.1371/journal.pone.0270352 (PMC9232155; doi:10.1371/journal.pone.0270352)
Supplement: Attachment — Submitted filename: Bonnet-Bayram_Reponse-reviewers.docx [file pone.0270352.s002.docx]

**Reviewer's Responses to Questions**

Dear Editor, and dear reviewer

We are thankful for having the opportunity to submit a revised draft of our manuscript. We appreciate the time and effort that you and the reviewers have dedicated to assessing our manuscript and are grateful for the insightful comments and feedback you provided. We are very grateful with the reviewer for their detailed and meticulous review of our manuscript. We modified the manuscript according to their suggestions, and reviewed the manuscript at length at their suggestion. These changes are highlighted within the manuscript in red.

Please, find a point-by-point response to the reviewers’ comments below.

Sincerely,
Marcela Perrone-Bertolotti for all of the authors

**Reviewer #1**: I find the paper very interesting and well written. In two different experiments, it has been shown the impact of motor imagery on lexical-semantic access, specifically it has been demonstrated that a kinesthetic motor imagery training improves language comprehension

We appreciate the time and effort that the reviewer dedicated to assessing our manuscript and are grateful for the insightful comments and feedback they provided.

Please find below my comments:

Introduction
The introduction lack of some references about behavioural and neurophysiological studies on the cross-talk between action and language, which sometimes are in contrast. Indeed some studies have highlighted a facilitation, some others an interference effect in case of congruency between the meaning of the sentence and the performed action.

I suggest to look at:

Scorolli, C., & Borghi, A. M. (2007). Sentence comprehension and action: Effector specific modulation of the motor system. Brain research, 1130, 119-124.
Gianelli, C., & Dalla Volta, R. (2015). Does listening to action-related sentences modulate the activity of the motor system? Replication of a combined TMS and behavioral study. Frontiers in psychology, 5, 1511.
Sato, M., Mengarelli, M., Riggio, L., Gallese, V., & Buccino, G. (2008). Task related modulation of the motor system during language processing. Brain and Language, 105(2), 83–90.
Miller, J., Brookie, K., Wales, S., Wallace, S., & Kaup, B. (2018). Embodied cognition: Is activation of the motor cortex essential for understanding action verbs?. Journal of Experimental Psychology: Learning, Memory, and Cognition, 44(3), 335.
Chersi, F., Thill, S., Ziemke, T., & Borghi, A. M. (2010). Sentence processing: linking language to motor chains. Frontiers in neurorobotics, 4, 4.
The authors does not mention any reference concerning the relation between action and abstract/concrete language, although such dichotomy is matter of investigation of the studies and formulate some predictions.
Please look at:
Scorolli, C., Binkofski, F., Buccino, G., Nicoletti, R., Riggio, L., & Borghi, A. M. (2011). Abstract and concrete sentences, embodiment, and languages. Frontiers in Psychology, 2, 227.
Ghandhari, M., Fini, C., Da Rold, F., & Borghi, A. M. (2020). Different kinds of embodied language: A comparison between Italian and Persian languages. Brain and Cognition, 142, 105581.
Desai, R. H., Conant, L. L., Binder, J. R., Park, H., & Seidenberg, M. S. (2013). A piece of the action: modulation of sensory-motor regions by action idioms and metaphors. NeuroImage, 83, 862-869.
Sakreida, K., Scorolli, C., Menz, M. M., Heim, S., Borghi, A. M., & Binkofski, F. (2013). Are abstract action words embodied? An fMRI investigation at the interface between language and motor cognition. Frontiers in human neuroscience, 125.
Scorolli, C., Binkofski, F., Buccino, G., Nicoletti, R., Riggio, L., & Borghi, A. M. (2011). Abstract and concrete sentences, embodiment, and languages. Frontiers in Psychology, 2, 227.

Thank you for highlighting this point and for providing us with additional references. In agreement with your comment, the following changes have been made to the introduction in efforts to explain the motor cortex’s involvement in the processing of abstract language:

*Line 73-93:*

*The implication of the motor cortex in concrete action-verb processing is frequently compared to its implication in the processing of abstract verbs (i.e., without a specific action representation, such as “to accept”). Although the role of motor activation in abstract-concept processing remains to be defined, studies have shown that the processing of abstract words is likely to involve motor regions, yet to a lesser extent than concrete language. For instance, when asked to read sentences varying in their level of concreteness (i.e., from purely concrete to merely abstract), results from the fMRI study of Sakreida et al. (2013) indicated that all sentences elicited the activation of core areas of the sensorimotor neural network, independently of their level of concreteness. However, the magnitude of these activation was greater for concrete sentences and specific activation in the language processing system were additionally found for abstract sentences (Sakreida et al., 2013; for a review, see Montifinese, 2019). A similar pattern of results was documented in TMS studies. For instances, Reilly and colleagues (2019) showed that the stimulation of the primary motor cortex (M1) at 300 ms after the verb presentation impairs the comprehension of literal and metaphoric action (i.e., conveying abstract language) sentences, suggesting that M1 is involved in abstract-sentence processing. Hence, results from neuroimaging studies suggest that both abstract and concrete language processing elicit sensorimotor areas’ activation. However, a greater activation of motor areas in response to concrete language processing has been put forward, which is corroborated by behavioral studies indicating that concrete language processing is faster than abstract processing (Alyahya et al., 2018; Huang & Federmeier, 2015).*

In addition, we revised the introduction section to emphasize the contrast between facilitation and interference effects:

Line 54-72:

*For instance, reading or listening to verbs like “kick” or “grasp” induces leg and hand sensorimotor activation, respectively. Consistent with the embodied cognition framework, authors have hypothesized that these motor activations might facilitate action language processing. Results from studies using Transcranial Magnetic Stimulation (TMS) have supported this hypothesis by showing that TMS applied to the leg and arm motor area, while participants are asked to perform a lexical decision on leg-related and arm-related action verbs, specifically reduces the processing time when the stimulation site matches the verb’s effector (Pulvermüller et al., 2005). On the contrary, inhibitory stimulation induced by repetitive-TMS has been shown to increase the processing time of the actions associated with the represented effector (Repetto et al., 2013). Additional evidence has been provided by electrophysiological studies indicating that these motor activations arise as early as 200 ms following verb presentation, suggesting that the motor system might play a facilitatory role in lexical-semantic processing (1,10). However, results from behavioral experiments are somewhat less straightforward. Indeed, although the action-sentence compatibility effect is well documented in the literature, with results showing a facilitation of action-language processing in congruent language-effector contexts (Andres, Finocchiaro, Buiatti, & Piazza, 2015; Boulenger et al., 2006; Chersi et al., 2010; Sato et al., 2008), some studies reported an opposite pattern of results, therefore pointing towards an interference effect (Aravena et al., 2012 , also see Morey et al, 2021 for a multi-lab ACE non-replication).*

These additions were also incorporated in the Discussions:

Discussion of Experiment 1:

Lines 501-502:

*This facilitation effect could be explained by a motor pre-activation induced by KMI but not by SVI.*

Lines 511-519:

*Moreover, the main effect of concreteness—resulting in shorter RTs for concrete items than for abstract items—supports previous results and can be explained by a difference in imageability between the two types of items (i.e., concrete words being highly-imageable, while abstract words are low-imageable, Alyahya et al., 2018; Huang & Federmeir, 2015; Wiemer-Hastings & Xu, 2005). Finally, results on the semantic categorization task did not reveal any processing differences across item types, which suggests that the potentially beneficial effect of KMI training on language comprehension is not limited to the specific trained items but is able to extend to other verbs.*

Lines 521-535:

*Different explanatory hypotheses can be put forward in light of the results. One way to explain the interaction effect that resulted in a greater training effect on abstract verbs for the KMI group revolves around a mostly beneficial effect of KMI training for words that are more difficult to process and less imageable. However, this explanation might be unlikely as results from the sentence-picture matching task only partially support our hypothesis, yet this task was maximizing degraded speech perception, which should have increased the difficulty associated with language processing. Conversely, it could be that the KMI training, and the consequent motor-cortex activation, resulted rather in an interference effect than a facilitation during the processing of action verbs, especially considering the effects of context on affordance activation. For instance, tasks and their different stimuli lead to different activations depending on the context and on the goal of the participant (Mazzuca et al., 2021). Affordances also vary depending on ownership (Mazzuca et al., 2021), and the stimuli used in the sentence-picture matching task all included sentences referring to a third person represented in the pictures, which rather rely on the comprehension of action and action goals of others (Mazzuca et al., 2021).*

Discussion of Experiment 2:

Lines 790-798:

*As discussed in Experiment 1, it is important to consider that the sentence-picture matching task featured a third-person individual executing an action or illustrating an abstract concept, concepts which are usually low-imageable (Alyahya et al., 2018; Huang & Federmeir, 2015). The illustration of a third person executing an action may have activated affordances different from those activated by the KMI training, in which participants are asked to perform first-person imagery. This may have resulted in an interference effect for the KMI participants (Mazzuca et al., 2021), while the SVI training may have indirectly facilitated the imageability of abstract concepts presented in the sentence-picture matching task through visual imagery skills improvement.*

Lines 800-803:

*Moreover, the results in this second experiment showed that more improvement was observed for abstract items compared to action items. The latter finding possibly indicated that our action items required relatively less effort compared to abstract items, as previously shown in Experiment 1.*

General Discussion:

Lines 846-852:

*Furthermore, as observed in previous studies (19,69), it seems that abstract items might be harder to process, potentially due to the high-imageability of concrete items as compared to low-imageable abstract items. It is interesting to note that some experiments show that the motor system is more involved in language processing when the processing is considered difficult (78–80). Thus, the interaction that we observed may be explained by greater involvement of the motor system in the processing of abstract verbs.*

Sample size:
The authors does not justify with any reference to the literature or power analysis the sample size of the both the experiments. Please provide a justification.

Since there were no previous studies examining the effect of a KMI training on language comprehension performances, we did not run a-priori sample size estimations. However, note that Locatelli et al. (2012) used a similar design as the one being proposed in the present series of experiments (i.e., manual training and pre-post training assessment of language comprehension) on a sample size of 10 participants per group. In Experiment 1, which included only a post-training language assessment, our sample size was almost twice as great as the one in Locatelli et al.’s (18 participants per group). In addition, with a total sample size of 53 participants in Experiment 2, our sample size greatly exceeded the one proposed in the study of Locatelli et al.

Following your comment, we decided to provide effect sizes and a-posteriori analysis of observed power for all the significant effects, included in the results section of both experiments.

Could you please report and translate the items in English of KIVQ-10?

We added the following elements to the Method section of Experiment 1. These are the original translations by the original authors Malouin et al. (2007), lines 243 *-255:*

*The 10-item KVIQ-10 was included as a measure of participants’ ability to visualize and feel imagined movements. In this questionnaire, participants were asked to execute a movement once, then imagine themselves doing it while focusing on the intensity of the kinesthetic sensations elicited by the imagery (kinesthetic subscale), or on the visual clarity of the imagery (visual subscale). The movements that participants executed and then imagined were forward shoulder flexion, thumb-fingers opposition, forward trunk flexion, hip abduction, and foot tapping. On the visual subscale, they rated their imagery as “5 = Image as clear as seeing”, “4 = Clear image”, “3 = Moderately clear image”, “2 = Blurred image”, or “1 = No image”. On the kinesthetic subscale, they rated their kinesthetic sensations as “5 = As intense as executing the action”, “4 = Intense”, “3 = Moderately intense”, “2 = Mildly intense”, or “1 = No sensation”. Participants were also asked to perform a mental chronometry task(60), which measured the temporal correlation between real and imagined movements.*

Lines 214: “presented” repeated twice

The manuscript was amended accordingly.

Why did you opt for a between-subjects design in the first experiment, instead of a within-subject design in which participant would have performed in different days?

Given that the impact of motor imagery on language comprehension is a quite new area of research, it is difficult to anticipate the duration of the effects of such MI training. In the physical practice domain however, Ruffino et al. (2019) showed that a single MI-training session was able to induce use-dependent plasticity, thus suggesting that an acute MI-training session might lead to potential long-lasting effects. Hence, by using a within-subject design in which participants would have been exposed to the two types of training over different days, we would have been able to interpret the results from the first training session. However, as we cannot predict the duration of the effects of this first session, we would have been unable to disentangle what part of the results could be attributed to the training that participants had just been exposed to from the long-lasting effects of the training received during the first session (or even, the interaction of the two training types). To accurately interpret our results with regards to a specific type of training (i.e., KMI vs. SVI), we have opted for the use of a between-subjects design.

Data Analysis
Could you please report the number of RTs discarded in both the experiments? .

The manuscript was amended accordingly in the corresponding Data analysis sections, specifically, in lines 436-437 and lines 727-728.

Could you explain the Cook D criteria?

As proposed by Cook and Weisberg (1982) in “Residuals and Influence in Regression”, observations with a Cook’s D value greater than 1 were considered as outliers. The manuscript was revised accordingly in lines 391-393:

*Observations with an SDR greater than 4 and/or a Cook’s D value greater than 1 were considered extreme and were thus excluded (66)*

Did you adopt as criteria the stepwise procedure in your models selection? Starting with the model with the more complex random structure? See the following reference:
Barr, D. J., Levy, R., Scheepers, C., & Tily, H. J. (2013). Random effects structure for confirmatory hypothesis testing: Keep it maximal. Journal of memory and language, 68(3), 255-278.

The data analysis section was amended as follows and the corresponding references were added, lines 392-393:

*Concerning RT analysis, models with maximal random effects structure (Barr et al., 2013; Bates et al., 2015) were adopted.*

Results & Discussion

I see that in the first experiment you got that participants were faster with abstract as compared with concrete verbs. Why in your opinion this is the case? Could you please argument more about that in the discussion?

Thank you for this suggestion. Accordingly, the revised manuscript now includes additional arguments pertaining to the differences in RTs depending on verb concreteness.

Lines 800-803:

*Moreover, the results in this second experiment showed that more improvement was observed for abstract items compared to action items. The latter finding possibly indicated that our action items required relatively less effort compared to abstract items, as previously shown in Experiment 1.*

Discussion of Experiment 2:

Lines 790-798:

*As discussed in Experiment 1, it is important to consider that the sentence-picture matching task featured a third-person individual executing an action or illustrating an abstract concept, concepts which are usually low-imageable (19,70). The illustration of a third person executing an action may have activated affordances different from those activated by the KMI training, in which participants are asked to perform first-person imagery. This may have resulted in an interference effect for the KMI participants (72), while the SVI training may have indirectly facilitated the imageability of abstract concepts presented in the sentence-picture matching task through visual imagery skills improvement.*

General Discussion:

Lines 846-852:

*Furthermore, as observed in previous studies (19,70), it seems that abstract items might be harder to process, potentially due to the high-imageability of concrete items as compared to low-imageable abstract items. It is interesting to note that some experiments show that the motor system is more involved in language processing when the processing is considered difficult (79–81). Thus, the interaction that we observed may be explained by greater involvement of the motor system in the processing of abstract verbs.*

Did you consider the influence of the culture in explaining your results? Were all your participants native French? Was someone bilingual?
I recommend to consider these factors as it is suggested in Ghandhari, M., Fini, C., Da Rold, F., & Borghi, A. M. (2020). Different kinds of embodied language: A comparison between Italian and Persian languages. Brain and Cognition, 142, 105581.

As explained in the Method section, our inclusion criteria referred only to having French as a first language (i.e., participants had to be native French speakers). Since we did not verify the presence of multilingualism among our participants, we cannot exclude the fact that a percentage of our sample may have more than one native language and/or have learned additional languages following French acquisition.

Nevertheless, we agree that culture is a factor worthy of discussion in our study and that multilingualism should be controlled for in future studies. Therefore, we expanded the discussion to include the following:

Lines 934-959:Finally, while our study included native French speakers only, we cannot disregard the potential role that bilingualism may have played. We did not examine whether any of our participants were early simultaneous bilingual (i.e., acquired French in parallel with a second native language), and influenced by another culture. Culture also appears to play a role in the extent of grounding of language. Ghandhari et al. (85) showed in their behavioral study that native Italian participants exhibited a facilitation effect when processing a concrete sentence that was congruent with a video displaying a movement, while native Persian participants exhibited an inhibition effect in the same congruent and concrete condition. Moreover, Italian participants were overall faster for concrete sentences (i.e., concreteness effect) regardless of congruency, whereas Persian participants presented inhibition particularly for concrete sentences in congruent trials, but processing times did not differ based on concreteness in incongruent trials (85). Likewise, in a TMS study comparing the semantic and motor resonance effects of first language versus second language, participants categorized verbs as abstract or concrete while TMS was applied to the hand motor cortex at varying time intervals following stimulus presentation. Results revealed that semantic resonance occurred at early stages of semantic processing (TMS applied at 125 ms post-stimulus) for first-language words but not second-language words, while motor resonance induced by TMS occurred at slightly later stages of semantic processing (275 ms post-stimulus) for second-language words. These results support the notion that action and language interact at early stages of word processing, and first-language and second-language are differently embodied (86). Nevertheless, studies show that children who acquire two languages from birth learn language in a manner comparable to monolingual children, whereas highly proficient late bilinguals seem to have a more grounded representation of their second language ((87) for a review).

It might depend on the level of integration between language and action, it might depend on the perceptual strength associated with the verbs.
One possibility is that abstract verbs “might occupy less the sensorimotor system “ and their categorization might be favoured, to better clarify , I mean that it might be possible that a congruency between action and concrete verbs is expressed by a interference effect instead of facilitation.

Thank you for pointing this out and for your valuable interpretation. We have, accordingly, incorporated detailed arguments regarding that matter in the discussion.

Discussion of Experiment 1:

Lines 501-502:

*This facilitation effect could be explained by a motor pre-activation induced by KMI but not by SVI.*

Lines 511-519:

*Moreover, the main effect of concreteness—resulting in shorter RTs for concrete items than for abstract items—supports previous results and can be explained by a difference in imageability between the two types of items (i.e., concrete words being highly-imageable, while abstract words are low-imageable (19,70,71). Finally, results on the semantic categorization task did not reveal any processing differences across item types, which suggests that the potentially beneficial effect of KMI training on language comprehension is not limited to the specific trained items but is able to extend to other verbs.*

Lines 521-535:

*Different explanatory hypotheses can be put forward in light of the results. One way to explain the interaction effect that resulted in a greater training effect on abstract verbs for the KMI group revolves around a mostly beneficial effect of KMI training for words that are more difficult to process and less imageable. However, this explanation might be unlikely as results from the sentence-picture matching task only partially support our hypothesis, yet this task was maximizing degraded speech perception, which should have increased the difficulty associated with language processing. Conversely, it could be that the KMI training, and the consequent motor-cortex activation, resulted rather in an interference effect than a facilitation during the processing of action verbs, especially considering the effects of context on affordance activation. For instance, tasks and their different stimuli lead to different activations depending on the context and on the goal of the participant (72). Affordances also vary depending on ownership (72), and the stimuli used in the sentence-picture matching task all included sentences referring to a third person represented in the pictures, which rather rely on the comprehension of action and action goals of others (72).*

Discussion of Experiment 2:

Lines 790-803:

*As discussed in Experiment 1, it is important to consider that the sentence-picture matching task featured a third-person individual executing an action or illustrating an abstract concept, concepts which are usually low-imageable (19,70). The illustration of a third person executing an action may have activated affordances different from those activated by the KMI training, in which participants are asked to perform first-person imagery. This may have resulted in an interference effect for the KMI participants (72), while the SVI training may have indirectly facilitated the imageability of abstract concepts presented in the sentence-picture matching task through visual imagery skills improvement.*

Lines 800-803:

*Moreover, the results in this second experiment showed that more improvement was observed for abstract items compared to action items. The latter finding possibly indicated that our action items required relatively less effort compared to abstract items, as previously shown in Experiment 1.*

General Discussion:

Lines 846-852:

*Furthermore, as observed in previous studies (19,70), it seems that abstract items might be harder to process, potentially due to the high-imageability of concrete items as compared to low-imageable abstract items. It is interesting to note that some experiments show that the motor system is more involved in language processing when the processing is considered difficult (79–81). Thus, the interaction that we observed may be explained by greater involvement of the motor system in the processing of abstract verbs.*

Finally, Here in you can find a broad perspective on the relation between the motor system and the language, especially the abstract one. Mazzuca, C., Fini, C., Michalland, A. H., Falcinelli, I., Da Rold, F., Tummolini, L., & Borghi, A. M. (2021). From affordances to abstract words: The flexibility of sensorimotor grounding. Brain Sciences, 11(10), 1304.

Results of Experiment 1 showed that participants were overall faster for concrete verbs compared to abstract verbs. To our knowledge, and as discussed in our General Discussion, abstract words were found to be more difficult to process compared to concrete or action words, possibly due to the high imageability of “concrete” words, such as action words (Alyahya et al., 2018), sometimes referred to as the “concreteness effect” (Huang & Federmeier, 2015). Participants in the KMI group were faster in the processing of abstract verbs, compared to concrete, action verbs. It could be because KMI training is beneficial for more difficult tasks or verbs.

We also took into consideration your suggestion about abstract verbs occupying less of the sensorimotor system, translating into an interference rather than a facilitation effect.

Discussion of Experiment 1:

Lines 521-535:

*Different explanatory hypotheses can be put forward in light of the results. One way to explain the interaction effect that resulted in a greater training effect on abstract verbs for the KMI group revolves around a mostly beneficial effect of KMI training for words that are more difficult to process and less imageable. However, this explanation might be unlikely as results from the sentence-picture matching task only partially support our hypothesis, yet this task was maximizing degraded speech perception, which should have increased the difficulty associated with language processing. Conversely, it could be that the KMI training, and the consequent motor-cortex activation, resulted rather in an interference effect than a facilitation during the processing of action verbs, especially considering the effects of context on affordance activation. For instance, tasks and their different stimuli lead to different activations depending on the context and on the goal of the participant (Mazzuca et al., 2021). Affordances also vary depending on ownership (Mazzuca et al., 2021), and the stimuli used in the sentence-picture matching task all included sentences referring to a third person represented in the pictures, which rather rely on the comprehension of action and action goals of others (Mazzuca et al., 2021).*

Discussion of Experiment 2:

Lines 790-803:

*As discussed in Experiment 1, it is important to consider that the sentence-picture matching task featured a third-person individual executing an action or illustrating an abstract concept, concepts which are usually low-imageable (Alyahya et al., 2018; Huang & Federmeir, 2015). The illustration of a third person executing an action may have activated affordances different from those activated by the KMI training, in which participants are asked to perform first-person imagery. This may have resulted in an interference effect for the KMI participants (Mazzuca et al., 2021), while the SVI training may have indirectly facilitated the imageability of abstract concepts presented in the sentence-picture matching task through visual imagery skills improvement.*

Lines 800-803:

*Moreover, the results in this second experiment showed that more improvement was observed for abstract items compared to action items. The latter finding possibly indicated that our action items required relatively less effort compared to abstract items, as previously shown in Experiment 1.*

General Discussion:

Lines 846-852:

*Furthermore, as observed in previous studies (53), it seems that abstract items might be harder to process, potentially due to the high-imageability of concrete items as compared to low-imageable abstract items. It is interesting to note that some experiments show that the motor system is more involved in language processing when the processing is considered difficult (54–56). Thus, the interaction that we observed may be explained by greater involvement of the motor system in the processing of abstract verbs.*

**Again, we thank you for your contributions and the references you introduced us to. They are a valuable addition to the literature we have reviewed, and to the article in question.**

**Reviewer #2:** The manuscript entitled "Kinesthetic Motor-Imagery Training Improves Performance on Lexical-Semantic Access" investigates the effect of Kinesthetic Motor-Imagery training on semantic processing. I very much enjoyed reading this manuscript and I believe that the research presented here would fit well within the scope of this journal. Within an embodied framework, the topic is timely. However the theoretical motivations could be better justified and the analytical scaffolding would benefit from a more thorough consideration of the literature.

We thank you for the time you took reading our manuscript. Thank you for the careful and meticulous remarks you left us, they helped us improve our work.

Major Remarks

Introduction
Overall, the theoretical motivation behind this study, as well as the theoretical implications, could be made more explicit. The introduction would benefit from an additional discussion on to the action-word processing (and possibly learning) literature. This would enrich the theoretical background and better justify the motivation for this work.

Thank you for highlighting this point and for contributing to the improvement of our article. Integrating your suggestions along with those of the other reviewer, we have revised the introduction section accordingly to emphasize the contrast between facilitation and interference effects reported in the action-word processing literature and dedicated a specific part of the introduction to the distinction between abstract and concrete language and their relation to action.

Lines 52-72:

*To illustrate, neuroimaging studies have revealed that action-related language processing elicits activation in motor and premotor brain regions (1–7), in a somatotopic manner (1) . For instance, reading or listening to verbs like “kick” or “grasp” induces leg and hand sensorimotor activation, respectively. Consistent with the embodied cognition framework, authors have hypothesized that these motor activations might facilitate action language processing. Results from studies using Transcranial Magnetic Stimulation (TMS) have supported this hypothesis by showing that TMS applied to the leg and arm motor area, while participants are asked to perform a lexical decision on leg-related and arm-related action verbs, specifically reduces the processing time when the stimulation site matches the verb’s effector (8). On the contrary, inhibitory stimulation induced by repetitive-TMS has been shown to increase the processing time of the actions associated with the represented effector (9). Additional evidence has been provided by electrophysiological studies indicating that these motor activations arise as early as 200 ms following verb presentation, suggesting that the motor system might play a facilitatory role in lexical-semantic processing (1,2,4,9). However, results from behavioral experiments are somewhat less straightforward. Indeed, although the action-sentence compatibility effect is well documented in the literature, with results showing a facilitation of action-language processing in congruent language-effector contexts (4,10–12), some studies reported an opposite pattern of results, therefore pointing towards an interference effect (13,14).*

Lines 73-93:

*The implication of the motor cortex in concrete action-verb processing is frequently compared to its implication in the processing of abstract verbs (i.e., without a specific action representation, such as “to accept”). Although the role of motor activation in abstract-concept processing remains to be defined, studies have shown that the processing of abstract words is likely to involve motor regions, yet to a lesser extent than concrete language. For instance, when asked to read sentences varying in their level of concreteness (i.e., from purely concrete to merely abstract), results from the fMRI study of Sakreida et al. (2013) indicated that all sentences elicited the activation of core areas of the sensorimotor neural network, independently of their level of concreteness. However, the magnitude of these activation was greater for concrete sentences and specific activation in the language processing system were additionally found for abstract sentences (Sakreida et al., 2013; for a review, see Montifinese, 2019). A similar pattern of results was documented in TMS studies. For instances, Reilly and colleagues (2019) showed that the stimulation of the primary motor cortex (M1) at 300 ms after the verb presentation impairs the comprehension of literal and metaphoric action (i.e., conveying abstract language) sentences, suggesting that M1 is involved in abstract-sentence processing. Hence, results from neuroimaging studies suggest that both abstract and concrete language processing elicit sensorimotor areas’ activation. However, a greater activation of motor areas in response to concrete language processing has been put forward, which is corroborated by behavioral studies indicating that concrete language processing is faster than abstract processing (Alyahya et al., 2018; Huang & Federmeier, 2015).*

Lines 57-72:

*Results from studies using Transcranial Magnetic Stimulation (TMS) have supported this hypothesis by showing that TMS applied to the leg and arm motor area, while participants are asked to perform a lexical decision on leg-related and arm-related action verbs, specifically reduces the processing time when the stimulation site matches the verb’s effector (Pulvermüller et al., 2005). On the contrary, inhibitory stimulation induced by repetitive-TMS has been shown to increase the processing time of the actions associated with the represented effector (Repetto et al., 2013). Additional evidence has been provided by electrophysiological studies indicating that these motor activations arise as early as 200 ms following verb presentation, suggesting that the motor system might play a facilitatory role in lexical-semantic processing (1,10). However, results from behavioral experiments are somewhat less straightforward. Indeed, although the action-sentence compatibility effect is well documented in the literature, with results showing a facilitation of action-language processing in congruent language-effector contexts (Andres, Finocchiaro, Buiatti, & Piazza, 2015; Boulenger et al., 2006; Chersi et al., 2010; Sato et al., 2008), some studies reported an opposite pattern of results, therefore pointing towards an interference effect (Aravena et al., 2012 , also see Morey et al, 2021 for a multi-lab ACE non-replication).*

Given that the authors are interested in the effect of training involving the motor system on language processing, I would suggest they discuss studies using language learning paradigms to test the influence of encoding language with action or while stimulating or inhibiting motor areas. For instance, applying TMS in motor areas in conjunction with hand action-based word learning showed a causal involvement of the motor cortex in action word acquisition and even indicated rapid wide-spread microstructural changes in the language system linked to the motor cortex function (Vukovic & Shtyrov, 2019; Vukovic et al., 2021). In another TMS study, Mathias and colleagues (2021) examined training on foreign language words with sensorimotor (gestures) or visual (pictures) enrichment. They measured learning using a translation task while either repetitive TMS was applied bilaterally over the primary motor cortex, or a sham stimulation was applied. Perturbation from repetitive TMS lead to slower translations of sensorimotor-enriched words but not sensory-enriched words.There are also several fMRI studies that look at encoding while observing or performing actions or gestures (Fargier et al., 2012, Macedonia et al., 2019; Macedonia & Mueller, 2016).

We have carefully read the study by Vukovic & Shtyrov (2017) and found it to be an essential addition to our bibliography, as well as an inspiration for future studies involving our KMI training. Specifically, we found it to be a study inspiring the implementation of our protocol in the acquisition of foreign languages. To test the role of the primary motor cortex in the encoding process of aspects of action verbs and object nouns, Vukovic & Shtyrov (2019) delivered theta-burst stimulation to groups of participants before they learned new words in three TMS conditions. TMS delivered to M1, as compared to active control and sham control TMS, interfered with learning as illustrated by kinematic and behavioral measures. These results illustrate a causal role of M1 in the word-learning process, as disruption of M1 directly and negatively affected the learning process. Results further revealed a category-specific effect, whereby in a lexical decision task, RTs differed based on word categories. Specifically, only the processing of action-related words was influenced by TMS. The overall pattern of results highlights M1’s involvement in early phases of word learning and encoding. The study clearly showed that the motor system is specifically implicated in the encoding and representation of sensorimotor concepts. Mathias and colleagues (2021) employed an rTMS protocol involving a sensorimotor-enriched training for foreign-language learning. The words presented in the training were accompanied by complementary gestures (i.e., sensorimotor-enriched training) or pictures (sensory-enriched). The task required participants to translate the newly-acquired words while they were stimulated with rTMS to M1. This stimulation slowed down the translation process as compared to sham for sensorimotor-enriched foreign words, but not sensory-enriched foreign words. The results may show that the sensorimotor-enriched training induced changes in the representations of foreign words in M1, explaining why rTMS affected the translation process of these words only.

However, we have decided to not include the study by Vukovic & Shtyrov (2019) or Mathias et al. (2021) in our introduction or discussion. We already made extensive edits to our manuscript (referenced in other sections) and added references suggested by both reviewers to enrich our introduction as well as discussion. Nonetheless, we thank you for the compelling and valuable resources you have suggested.

One of the authors’ hypotheses involves a contrast between abstract and action verbs (i.e., that the KMI group will perform better on action compared to abstract verbs). As such, the introduction would benefit from a section on the contrast between abstract and concrete language to justify this hypothesis. Some argue that abstract and concrete are represented differently (i.e., verbal-language vs. perceptual) (Montefinese, 2019; Wang et al., 2010; but see Ponari et al 2018). In addition, it has been shown that concrete words are processed more quickly than abstract words (for a review, see Huang & Federmeier, 2015), sometimes referred to as the concreteness effect.

Integrating your suggestions together with those of the second reviewer, the introduction has been expanded as follows to emphasize the dichotomy between abstract and action verbs:

Lines 73-93:

*The implication of the motor cortex in concrete action-verb processing is frequently compared to its implication in the processing of abstract verbs (i.e., without a specific action representation, such as “to accept”). Although the role of motor activation in abstract-concept processing remains to be defined, studies have shown that the processing of abstract words is likely to involve motor regions, yet to a lesser extent than concrete language. For instance, when asked to read sentences varying in their level of concreteness (i.e., from purely concrete to merely abstract), results from the fMRI study of Sakreida et al. (2013) indicated that all sentences elicited the activation of core areas of the sensorimotor neural network, independently of their level of concreteness. However, the magnitude of these activation was greater for concrete sentences and specific activation in the language processing system were additionally found for abstract sentences (Sakreida et al., 2013; for a review, see Montifinese, 2019). A similar pattern of results was documented in TMS studies. For instances, Reilly and colleagues (2019) showed that the stimulation of the primary motor cortex (M1) at 300 ms after the verb presentation impairs the comprehension of literal and metaphoric action (i.e., conveying abstract language) sentences, suggesting that M1 is involved in abstract-sentence processing. Hence, results from neuroimaging studies suggest that both abstract and concrete language processing elicit sensorimotor areas’ activation. However, a greater activation of motor areas in response to concrete language processing has been put forward, which is corroborated by behavioral studies indicating that concrete language processing is faster than abstract processing (Alyahya et al., 2018; Huang & Federmeier, 2015)..

p*. 6 It would be helpful for the reader to briefly define motor imagery and to justify using motor imagery as opposed to action. Is the idea to show that this motor-to-semantic priming is robust and hence “works” with motor imagery and not just overt action?

We have duly incorporated a brief definition of motor imagery and have further justified why we believe that motor imagery offers broader opportunities for clinical application than physical practice.

Lines 126-137:

*If sensorimotor expertise—acquired through action observation and action execution (35,37)—can modify the motor system and its connections to the language system, we assume that motor imagery (i.e., the mental simulation of an action without concomitant movement production, (38) may also induce changes in the motor system and its functional connectivity. Indeed, the existence of an overlap in brain activation during motor imagery (MI), actual action execution and action observation (39) have been well established, suggesting that these different processes share similar motor representations (27,40). Specifically, neuroimaging studies revealed that imagined and executed movements share common underlying neural networks, including structures such as the basal ganglia, cerebellum, primary motor and premotor cortices (particularly the supplementary motor area), inferior parietal cortex, prefrontal cortex (38,39,41–43).*

Lines 157-174:

*In conclusion, several studies highlighted that MI increases corticospinal excitability and engages motor regions that are involved during action-language processing. In addition, studies evaluating the neural correlates of MI, showed that MI, action execution, and observation involve a common brain network. They also showed that action execution and observation induce improvement in language comprehension (e.g., 20,22). Given the arguments presented above, we hypothesized that imagining the execution of movements might also improve language processing. From a clinical perspective and in comparison, with action execution training, MI-based training has the advantage that it can be used in populations with limited motor functioning, and requires little-to-no physical exertion.*

**Experiment 1**
It is not clear why the authors hypothesize that participants in the KMI group would perform better in both tasks; this should be specified/made explicit. The same is true for the hypothesis concerning the abstract/action verb distinction.

In order to clarify our hypotheses, the following paragraphs have been added to the revised manuscript.

Lines 198-204:

*Following the embodied view of language, which has proposed that language is grounded in action, we hypothesized that if a kinesthetic motor imagery training can indeed improve lexical-semantic access, better performances on both language tasks are to be expected from participants in the KMI group than from those in the SVI control group (given that both tasks are driven by lexical-semantic processing and even though the sentence-picture matching task requires an evaluation of visual-semantic similarities, Harvey & Schnur, 2016; Huang et al., 2017).*

Lines 204-216:

*In addition, if the proposed KMI training protocol can induce specific effects for the trained imagined movements only (i.e., simple motor-to-semantic priming), we expect to find greater differences between concrete and abstract verbs, and target versus non-target items in the KMI group compared to the SVI control group. However, in case the KMI training protocol can induce training transfer effects, the magnitude of the difference between concrete and abstract verbs in one hand, and target versus non-target items in the other hand might be substantially reduced in the KMI group. The hypothesized underlying mechanisms rely in a spread of motor activations (i.e., most likely primary motor areas) along with neuroplasticity effects if the training expand to non-target items only; or larger spread of activations to sensorimotor areas (i.e., most likely higher-order motor areas in addition to primary sensorimotor ones) along with neuroplasticity effects, if transfer effects are not only noticed on non-target items, but also on abstract ones.*

Moreover, your comment prompted us to further examine the cognitive processes at stake in the sentence-picture matching tasks, which led us to modify the discussion section as follows:

Lines 874-897:

*Some of the results may in fact be driven by the demands of the sentence-picture matching task. It could be due to the affordances activated by the verbs in the sentences, by the actions depicted in the picture, or by the contextual elements in the pictures (Mazzuca et al., 2017). Nevertheless, while the sentence-picture matching task does involve semantic retrieval (Harvey & Schnur, 2016; Huang et al., 2017), it also may induce semantic interference in participants when pictures and words belong to the same semantic category (Harvey & Schnur, 2016). The present KMI protocol may have activated semantic representations of body parts related to the actions verbs, which may have exacerbated this interference effect, as semantic-to-motor priming has been documented (Boulenger et al., 2020). In our study, we used sentences rather than words, but the sentences were controlled on various psycholinguistic variables and trial responses revolved around the verb in the sentence. Indeed, embedding verbs in sentences may complicate a sentence-picture matching task compared to words only, as they require accessing additional semantic orders (Huang et al., 2017). The semantic categorization task requires access to semantic information about semantic membership but does not necessarily require access to fine-grained semantic level categories (Harvey & Schnur, 2016). By contrast, sentence-picture matching tasks necessitate the navigation of visual elements as well as making fine-grained semantic decisions about a set of visual elements that could be associated with the word, or sentence (Harvey & Schnur, 2016; Huang et al., 2017). It may also be that a visual imagery training may have facilitated the subsequent visual search required in the sentence-picture semantic task. In Experiment 2, a longer training was required, which may explain why the main effect of the group (more improvement for the SVI group) in the sentence-picture matching task was found in Experiment 2, while the opposite pattern of results (i.e., slower RTs for the SVI group) was found in Experiment 1, after an acute training session.*

**Methods**
In line 185 the authors wrote “We chose the KMI modality as it activates the motor neural network to a greater extent (42).” To a greater extent than what?

We clarified in the manuscript as follows:

Lines 272-274:

*We chose the KMI modality as it activates the motor neural network to a greater extent compared to visual MI (Guillot et al., 2007).*

For experiment 2 it is noted that “Each training session lasted about 15 minutes” but no duration is given for experiment 1.

Information about the duration of the training session was added for Experiment 1 as well.

Line 298:

*Each training (KMI and SVI) protocol lasted about 15 minutes.*

Again, for Experiment 2, it is hypothesized that participants would show greater improvement in processing action compared to abstract verbs. I’m assuming that the reason behind this is a belief that the motor cortex is more involved in representing abstract verbs but if this is the case, the story would be more coherent if it is stated in the general introduction or the Experiment introductions.

We had hypothesized that a motor-to-semantic priming would occur driven by neuroplastic effects induced by the KMI training. However, we did not previously consider the potential interference effects and incongruent affordances. Indeed, our hypotheses were initially based on studies showing that physical practice can improve language processing (e.g., Beilock et al., 2008; Locatelli et al., 2012) together with TMS studies showing that stimulating the motor cortex can facilitate access to action language. In addition, considering the limited number of stimuli in the language tasks (14 abstract verbs, 14 target action verbs, 14 non-target action verbs), we did not expect an interference effect to significantly affect the general pattern of results. As mentioned above in response to comment 5, we have now attempted to clarify our hypotheses. Finally, the current manuscript review process has brought several references to our attention, which helped us better interpret our results regardless of our previous hypotheses. These updated interpretations can be found in the manuscript as follows, in lines 898-910:

*It has been brought to our attention by the reviewers and the resources they provided that a KMI training, followed by tasks including some action verbs, may result with an interference rather than a facilitation of the processing of action words, due to the different afferences that the KMI training activates, as they may clash with the afferences activated by the action verbs (Mazzuca et al, 2021). This information was not included in our initial hypotheses, as we had previously based our hypotheses on studies showing that physical expertise and short manual trainings lead to an enhanced performance on language tasks (Beilock et al., 2008; Locatelli et al., 2012). We use this information now to better interpret the results of the second experiment, compared to the first one. In the first experiment, participants in the KMI group were overall faster than participants in the SVI group, and this difference was significantly greater for abstract verbs compared to concrete verbs in the semantic categorization task. In the second experiment, there were no interactions in the semantic decision task, and the SVI group had shorter RTs in the sentence-picture matching task.*
Discussion(s)

Line 385: “the results showed a significant main effect of the training group, suggesting that KMI training can facilitate lexical-semantic processing. This facilitation effect could be explained by a motor pre-activation induced by KMI but not by SVI.”

A semantic-to-motor priming effect, but also interference when motor preparation and compatible semantic processing occur simultaneously, has been largely discussed in the Action-Sentence compatibility effect literature. The authors might want to include a few of these references in their introduction/discussion (Aravena et al., 2012; Borreggine, 2008; Boulenger et al., 2006; de Vega et al., 2013; Diefenbach et al., 2013; Dudschig et al., 2014; Glenberg and Kaschak 2002; Zwaan & Taylor, 2006). In this same context, it could be interesting to discuss García and Ibáñez’s HANDLE model (2016).

Thank you for raising this important point. Both introduction and discussion were revised to emphasize the importance of such interference effects in the action-language field. We have reviewed the references you have provided and found them to be useful in the interpretation of our results. At your suggestion, we have read Garcia and Ibáñez’s (2016) HANDLE model and found it to be a valuable and essential addition to our bibliography and a source of inspiration for future experiments. However, we decided not to include it in our present work, as we already expanded our Introduction as well as our Discussions with references that the reviewers have brought to our attention. We did include some of the references you have hereby cited in the Introduction and Discussion as follows:

Introduction, lines 67-72:

*However, results from behavioral experiments are somewhat less straightforward. Indeed, although the action-sentence compatibility effect is well documented in the literature, with results showing a facilitation of action-language processing in congruent language-effector contexts (Andres, Finocchiaro, Buiatti, & Piazza, 2015; Boulenger et al., 2006; Chersi et al., 2010; Sato et al., 2008), some studies reported an opposite pattern of results, therefore pointing towards an interference effect (Aravena et al., 2012 , also see Morey et al, 2021 for a multi-lab ACE non-replication).*

General Discussion, lines 898-933:

*It has been brought to our attention by the reviewers and the resources they provided that KMI training, followed by tasks that include some action verbs, may result in an interference rather than a facilitation of the processing of action words (Mazzuca et al, 2021). In Experiment 1, participants in the KMI group were overall faster than participants in the SVI group, and this difference was significantly greater for abstract verbs compared to concrete verbs in the semantic categorization task. The participants in the KMI group were faster in the processing of target verbs compared to non-target, untrained verbs, possibly indicating that the KMI training interfered with the processing of the untrained action verbs specifically. In Experiment 2, there were no interactions within the semantic categorization task, and the SVI grouThe longer training protocol implemented in Experiment 2 could have induced neuroplasticity effects that affected performance on language comprehension in a different pattern from Experiment 1. Indeed, it has been shown that a single session of MI practice induces use-dependent plasticity, driven by an increase of corticospinal excitability in agonist muscles, and this excitability was shown to return to baseline levels when assessed 30 minutes later (50). In Experiment 1, the affordances activated by the KMI training may have been incongruent with the affordances activated by some of the action verbs included in the language tasks, namely, the sentence-picture matching task that included more contextual elements in its material. This might have been illustrated by the interaction effect observed, which was opposite to our previously-hypothesized interaction of a specific facilitatory effect of the KMI training in action verbs compared to abstract verbs. For the sentence-picture matching task, it seems that the absence of pre-activation resulted in an advantage for the SVI participants, who had shorter RTs compared to the KMI participants. Since there was still a main effect of the group in one task but not the other task, it lessens the likelihood that the effect observed in Experiment 1 was due to faster manual responses induced by KMI hand-action training. Moreover, abstract verbs seem to be grounded in sensorimotor areas, but to a lesser extent compared to action verbs (15) It could explain the interaction between group and verb type (i.e., target, non-target), as well as group and concreteness (i.e., abstract, concrete) of Experiment 1 in the semantic decision task, as abstract verbs did not induce interference, unlike untrained action verbs. Nevertheless, both facilitation and interference (or inhibition) effects highlight the bidirectional relationship between language and action.p demonstrated better improvement in reaction times in the sentence-picture matching task. It is possible that an interference occurred between the areas activated in the motor cortex, and the action-information conveyed by sentences and pictures in the sentence-picture matching task.*

Line 677 – “there are some factors not taken into consideration, such as gender and age” – are these important factors? Why? Predictions?

Age can reduce the vividness of imagery, while gender affects the timing of MI without impacting its vividness, whereby women perform slightly better when it comes to imagery timing (Subirats et al., 2018). However, our sample size was homogeneous in terms of age, with participants aged, on average, between 19 and 21 years, with a maximum SD = 2 years. As for gender, it is known to affect the timing of MI, an aspect of MI we measured in both experiments using the mental chronometry task, and before and after training in Experiment 2. In Experiment 1, results showed that participants in both groups did not differ in terms of mental chronometry. In Experiment 2, results showed that participants’ performance on mental chronometry did not improve from pre- to post-training.

Since our KMI training aimed to improve the kinesthetic aspect of MI, we had no predictions concerning the interaction between gender and performance on language tasks nor improvement on the KVIQ-10. As for age, we recruited university students and expected them to have similar ages. Moreover, we evaluated their baseline imagery abilities using the KVIQ-10 prior to the training session in both experiments and found no significant difference between the two groups.

We therefore decided to remove this point about gender and age from our Discussion.

Line 687 - “Some previous studies found that motor-based training protocols can improve abstract verb processing” – At first this seems to contradict the statement on line 659 “… but to our knowledge, there are no studies that directly examined the effect of MI training on language comprehension tasks.” I think it might be helpful to point out that the motor-based training protocols referred to in line 687 involve actual action as opposed to MI.

While other studies explored the effects of motor-based functions such as action observation, action execution, or motor imagery combined with one of the latter, our study was the first to examine the effect of kinesthetic motor imagery alone on subsequent performance on language tasks.

We removed the sentence you have cited and clarified in the Conclusion as follows, lines 961-967:

*The present study showed for the first time to our knowledge that a KMI-only training, known to increase sensorimotor activation, can facilitate lexico-semantic processing, alluding to the presence of functional links between sensorimotor and language areas. Previous studies have investigated the role of motor experience and action observation (e.g., (52)), or motor imagery combined with another motor function (53), while ours was the first, to our knowledge, to assess the role of a KMI-only training on language comprehension.*

737 – Another place to possibly mention the TMS study described above, under Introduction

We added other TMS references in the introduction section as follows, lines 52-72:

*Results from studies using Transcranial Magnetic Stimulation (TMS) have supported this hypothesis by showing that TMS applied to the leg and arm motor area, while participants are asked to perform a lexical decision on leg-related and arm-related action verbs, specifically reduces the processing time when the stimulation site matches the verb’s effector (Pulvermüller et al., 2005). On the contrary, inhibitory stimulation induced by repetitive-TMS has been shown to increase the processing time of the actions associated with the represented effector (Repetto et al., 2013). Additional evidence has been provided by electrophysiological studies indicating that these motor activations arise as early as 200 ms following verb presentation, suggesting that the motor system might play a facilitatory role in lexical-semantic processing (1,10). However, results from behavioral experiments are somewhat less straightforward. Indeed, although the action-sentence compatibility effect is well documented in the literature, with results showing a facilitation of action-language processing in congruent language-effector contexts (Andres, Finocchiaro, Buiatti, & Piazza, 2015; Boulenger et al., 2006; Chersi et al., 2010; Sato et al., 2008), some studies reported an opposite pattern of results, therefore pointing towards an interference effect (Aravena et al., 2012 , also see Morey et al, 2021 for a multi-lab ACE non-replication).*

Conclusion
761 – “it relies on the abilities of various patients’ – I’m not sure what this means, I think it requires clarification

In the Conclusion of the current version of the manuscript, we clarified this as follows in lines 978-982:

*Indeed, MI presents several advantages that make it worthy of consideration for a complementary rehabilitation method of language deficits. For example, by relying on their residual abilities, such as mental imagery practice, patients for whom covert sensorimotor abilities are affected, could improve their motor functioning while potentially activating the language system.*

**Minor Comments**

English
I would suggest that the writers have the manuscript proofread by a native English speaker as some of the wording is sometimes awkward, stilted or too informal.
Below is a short non-exhaustive list with examples

107 – “that had effects on language functions” ◊ that affected language functions
121, 121 “In a first step” “In a second step” ◊ “First,” “Following this,”. Step 1 and Step 2 can be added in parentheses, as they are for Step 3.
131 – “participants had to judge as fast as possible” ◊ were asked to judge as quickly as possible.
145 – “To be included, they had to be right-handed, native French speakers ….” ◊ Participants were right-handed, native French speakers.
152 – “After receiving an explanation of the procedures, participants…”
158 – “we told participants that they were…” – “participants were told…”
204/205 – “Participants had to imagine” ◊ Participants were asked to imagine.
206 – “After reminding the instructions” ◊After being reminded of the instructions
218: “that are related to an action trained in the MI training phase…” ◊ related to an action from (or presented during) the training phase
225-227 – “the” can be taken out (i.e., lexical frequency, number of homophones, number of letters…)
256 – “Following” ◊ Following this
258 – “The pictures stayed on the screen” ◊ The pictures remained on the screen
259 – “There were three categories of sentences” ◊ The sentences fell under three categories
260 – “Sentences that contained one of the 14 target verbs”
261 – “one of the non-target verbs”
367 – “neither” ◊ either
424 – “was supposed to” ◊ aimed at further recruiting
139/426/623/643 etc. – “more important” ◊ greater
433 – “To be included, they had to be” ◊ Participants were right-handed, native French speakers.
457 – Participants took part in four training sessions: the first one on the same day they completed the pre-training assessment and on the following three days.
486 – “Wrong” ◊ incorrect
550 – “a better” ◊ greater
567 – take out “Results in terms of”
598 – “would improve” ◊ improved
657 – Several studies have examined
663 – “The present study had several advantages”: In English, an advantage is generally in relation to something else so you could say “The present study had several advantages in relation to other studies in this area” and site these studies, if that’s the case.
669 – “The present study failed to include” ◊ The present study did not include
677 – “there are some factors not taken into consideration” ◊ other, important, factors were not taken into consideration
694 – “For the abstract verb” ◊ For abstract verbs
701 – “Improved significantly more” ◊ improved significantly
709 – take out “had”
716 – take out “some”
723 – “it would not be expected that modulations of the motor system” -◊ modulations in the motor system would not be expected to influence…
725 – “and it would not be expected for the KMI group to have performed …” ◊ and the KMI group would not have been expected to perform better…
746 – can facilitate
748 – take out “A”

Line 214 - repetition of the word “presented”
Parts of the Methods are in the future
Line 235 - “the 42 experimental verbs were presented twice”
Line 362 – and a total of… (no comma)
Line 363 - In terms of accuracy, participants
It might be the version I received by all the figures are a bit blurry (especially the scale numbers). Also, for figure 2, the grey on “SVI” for “Sentence-picture matching” is not as dark as the grey on the figure.
597 – “The second experiment” ◊ It
603 – concreteness. This is effect is marginally significant, indicating that◊ concreteness, indicating that
657 – This paragraph has several sentences starting with “The present study”, which is very repetitive

We are very grateful for the time and efforts you have dedicated in putting together this list with examples. All wording mistakes you pointed out have been corrected in the revised version of the manuscript. Furthermore, the manuscript has now undergone a careful proofreading process, which–we hope–will have been enough to correct remaining stylistic, wording, and grammatical errors in addition to those you had mentioned.

6. PLOS authors have the option to publish the peer review history of their article ([what does this mean?](https://journals.plos.org/plosone/s/editorial-and-peer-review-process#loc-peer-review-history)). If published, this will include your full peer review and any attached files.

If you choose “no”, your identity will remain anonymous but your review may still be made public.

**Do you want your identity to be public for this peer review?** For information about this choice, including consent withdrawal, please see our [Privacy Policy](https://www.plos.org/privacy-policy).

Reviewer #1: No

Reviewer #2: No

[NOTE: If reviewer comments were submitted as an attachment file, they will be attached to this email and accessible via the submission site. Please log into your account, locate the manuscript record, and check for the action link "View Attachments". If this link does not appear, there are no attachment files.]

While revising your submission, please upload your figure files to the Preflight Analysis and Conversion Engine (PACE) digital diagnostic tool, <https://pacev2.apexcovantage.com/>. PACE helps ensure that figures meet PLOS requirements. To use PACE, you must first register as a user. Registration is free. Then, login and navigate to the UPLOAD tab, where you will find detailed instructions on how to use the tool. If you encounter any issues or have any questions when using PACE, please email PLOS at [figures@plos.org](mailto:figures@plos.org). Please note that Supporting Information files do not need this step.
